# Antihypertensive treatment improves glycemic control in patients with newly diagnosed type 2 diabetes mellitus: A prospective cohort study

**DOI:** 10.3389/fendo.2022.935561

**Published:** 2022-09-09

**Authors:** Jung-Chi Li, Po-Chung Cheng, Chien-Nin Huang, Li-Fen Jian, Ying-Syuan Wu, Chih-Li Lin

**Affiliations:** ^1^ Department of Internal Medicine, Division of Cardiology, Wuri Lin Shin Hospital, Taichung, Taiwan; ^2^ Department of Internal Medicine, Division of Endocrinology and Metabolism, Changhua Christian Hospital, Changhua, Taiwan; ^3^ Department of Internal Medicine, Division of Endocrinology and Metabolism, Chung Shan Medical University Hospital, Taichung, Taiwan; ^4^ Institute of Medicine, Chung Shan Medical University, Taichung, Taiwan; ^5^ Department of Nursing, Changhua Christian Hospital, Changhua, Taiwan; ^6^ Department of Family Medicine, Changhua Christian Hospital, Changhua, Taiwan; ^7^ Department of Medical Research, Chung Shan Medical University Hospital, Taichung, Taiwan

**Keywords:** type 2 diabetes mellitus, hypertension, diabetes therapy, hyperglycemia, treatment efficacy

## Abstract

**Background:**

Type 2 diabetes mellitus (T2DM) is a chronic metabolic disorder involving progressive pancreatic dysfunction. A substantial proportion of patients with T2DM cannot achieve euglycemia despite pharmacologic therapy. Preceding clinical studies have shown that hypertension contributes to glucose dysregulation, and investigators in this study hypothesized that antihypertensive treatment may improve glycemic control in patients with T2DM.

**Methods:**

This prospective cohort study investigates the effect of adding the antihypertensive drug Amlodipine to standard diabetes therapy on serum glycosylated hemoglobin A1c (HbA1c) and lipid profile in patients with newly diagnosed T2DM. The study enrolled a total of 168 participants with newly diagnosed T2DM.

**Results:**

Recipients of additional antihypertensive drug Amlodipine demonstrated significantly lower serum HbA1c (6.62% vs. 7.01%, P = 0.01), systolic blood pressure (132 mm Hg vs. 143 mm Hg, P < 0.001), and diastolic blood pressure (78.9 mm Hg vs. 86.0 mm Hg, P <0.001) compared to recipients of standard diabetes therapy after 24 weeks.

**Conclusion:**

Antihypertensive treatment with Amlodipine in addition to standard diabetes therapy improves glycemic control in patients with T2DM and may be an appropriate option in people with diabetes and concomitant hypertension to help maintain euglycemia.

## Introduction

Diabetes is a metabolic disorder that substantially influences national health infrastructures (1). Impaired insulin secretion and resistance to insulin action are important determinants in the pathophysiology of T2DM (2). The healthcare burden of vascular complications in diabetes is enormous and occurs relatively early in the disease process (3). Importantly, stringent glycemic control can attenuate both microvascular and macrovascular complications as well as lower mortality rate in people with diabetes.

A large prospective study has shown that inadequately treated hyperglycemia increases the mortality rate in people with diabetes (4). Moreover, adequate glycemic control is associated with better physiologic function in patients with preexisting T2DM (5). Therefore, attaining euglycemia is especially important for people with diabetes not only to reduce disease-specific mortality but also to preserve quality of life.

Currently there are several classes of antidiabetic drugs available for the treatment of T2DM (6), with more pharmacologic agents being tailored for clinical use. However, diabetes is a progressive disorder involving pancreatic beta-cell dysfunction (7), which is further exacerbated by insulin resistance and toxic metabolites of glucose oxidation. Indeed, nearly half of patients with T2DM remain inadequately treated despite multiple antidiabetic drugs, and numerous patients require additional glucose-lowering medications over time (8). Thus, newer strategies may be necessary to enhance the efficacy of current treatment modalities for diabetes.

Epidemiologic evidence suggests that hypertension frequently precedes the development of T2DM (9). Diabetes and hypertension share common mechanistic pathways including activation of the renin-angiotensin system, oxidative stress, and proinflammatory cytokines (10). Importantly, insulin resistance is associated with chronic hypertension (11), and lowering of blood pressure levels can attenuate the development of overt diabetes in patients with glucose intolerance (12).

Evidence suggests that antihypertensive drugs including calcium channel blockers may improve glycemic control in animal models of T2DM (13). Moreover, a study of glucose homeostasis suggests that antihypertensive medications increase blood perfusion in skeletal muscles, which are the primary glucose-metabolizing tissues in humans (14). A small clinical study demonstrates that the antihypertensive drug Azelnidipine significantly reduced plasma levels of glucose after an oral glucose tolerance test (15).

Considering the link between hypertension and glucose metabolism, the investigators hypothesized that stringent blood pressure management with the antihypertensive medication Amlodipine may improve glycemic control in patients with T2DM. The primary endpoint of this study is the change in plasma glucose parameters after 24 weeks of pharmacologic intervention. Secondary outcomes include the effect of additional antihypertensive medication on lipid profile, blood pressure and urine albumin excretion.

## Materials and methods

### Participant selection

This prospective study surveyed patients visiting the endocrinology clinic for eligibility. Inclusion criteria were as follows: (1) adults over 21 years of age, (2) T2DM diagnosed within one month of study referral according to criteria of the American Diabetes Association, namely serum glycosylated hemoglobin A1c (HbA1c) level ≧ 6.5%, fasting plasma glucose (FPG) ≧ 126 mg/dL, 2-hour postprandial plasma glucose levels after 75-gram oral glucose tolerance test ≧ 200 mg/dL, or random plasma glucose levels ≧ 200 mg/dL with classic symptoms of diabetes (3) recipients of metformin monotherapy, (4) no concomitant use of antihypertensive drug at study recruitment, and (5) compliance with medications and dietary intervention.

Exclusion criteria were as follows: (1) recipients of any second-line antidiabetic drug in addition to metformin, (2) recipients of any antihypertensive medication prior to recruitment, (3) patients with hemoglobin disorders, chronic kidney disease, or thyroid dysfunction, (4) pregnancy, and (5) inability to comply with therapeutic intervention.

### Study protocol

Baseline information including age, body mass index, sex and blood pressure were recorded at diagnosis of T2DM prior to pharmacologic treatment. Blood tests for serum HbA1c, plasma lipid levels, and serum creatinine were performed after an 8-hour fast. All participants received nutrition consultation by diabetes educators in which daily recommended calorie intake, carbohydrate counting technique, and exercise recommendations were provided. Moreover, participants were advised to reduce salt intake to less than 2400 milligrams a day to control hypertension. Diabetes educators contacted participants once a week to check on medication compliance and dietary habits.

Thereafter, participants were divided into two therapeutic cohorts according to shared decision making between patients and their attending physicians. The control group received standard diabetes therapy including metformin and dietary intervention without any additional antihypertensive drug. The antihypertensive cohort received 5 milligrams of Amlodipine once daily in tandem with standard diabetes therapy.

Both cohorts continued their therapeutic regimens for the next 24 weeks. Then the participants returned for follow-up blood tests after an 8-hour fast. Blood pressure measurements were also performed at the clinic visit.

### Outcome measures

The primary outcome measure of this study was the change in serum HbA1c after completing 24 weeks of pharmacologic treatment. Secondary outcomes include changes in plasma lipid levels, blood pressure, and urinary albumin excretion after treatment.

### Statistical analysis plan

Power analysis indicated that a sample size of 75 participants in each group was necessary to detect a significant difference in glucose parameters with 80% statistical power. The therapeutic cohorts include all participants who received at least one dose of the study prescriptions. Blood tests and baseline data at diagnosis of T2DM were considered as the baseline. Outcome measures in this study were based on laboratory tests at the follow-up clinic appointment after 24 weeks of pharmacologic therapy.

The baseline characteristics and clinical outcomes of the treatment groups were compared using Student’s independent t-test for continuous variables and Pearson’s χ^2^-test for categorical variables. Furthermore, variables were compared to baseline levels using paired sample t-test to evaluate the efficacy of antidiabetic treatment. Multiple regression analysis was performed to identify clinical determinants of serum HbA1c levels. All statistical analysis was performed using IBM Statistical Package for the Social Sciences version 22.0 (IBM Statistics for Windows, New York, USA). A two-tailed P value of less than 0.05 indicated statistical significance.

## Results

**Figure 1:**
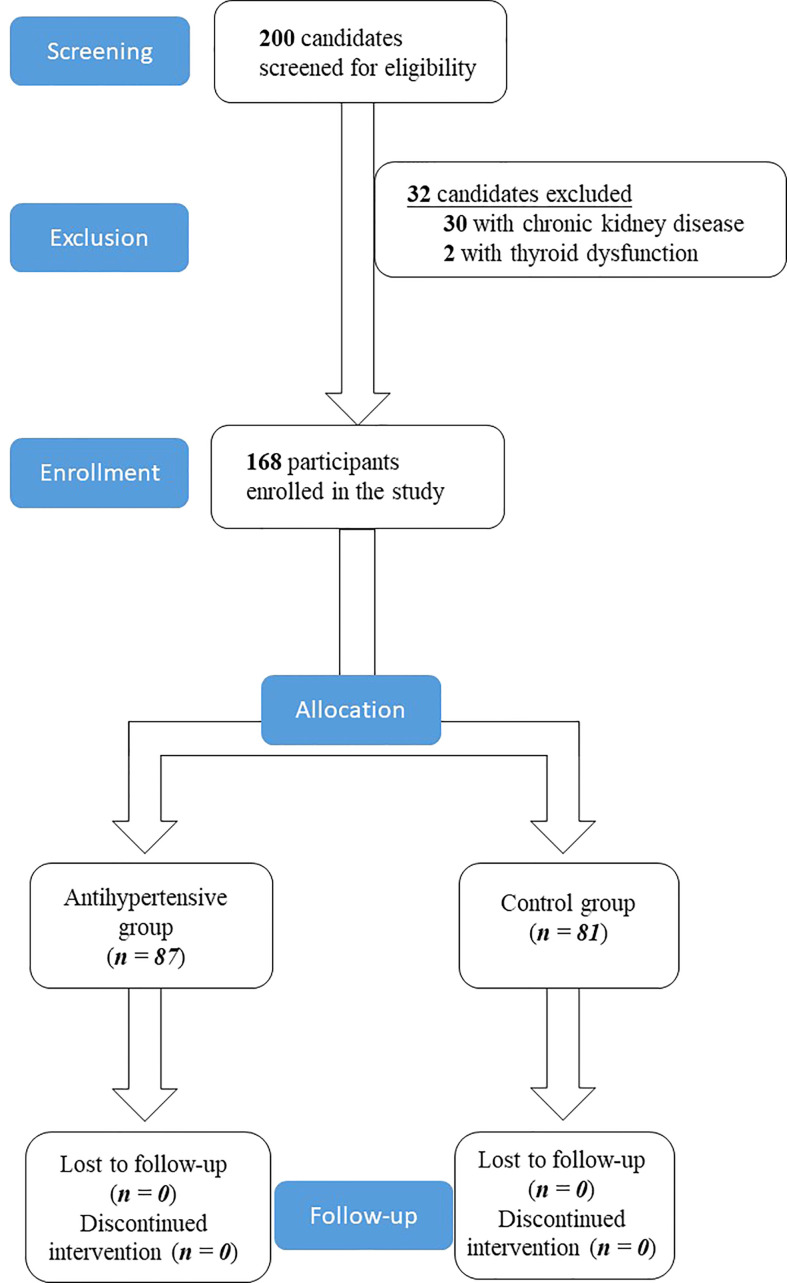
Enrolment protocol of the study.

The study screened 200 candidates visiting the endocrinology clinic for eligibility. Thirty patients were excluded due to chronic kidney disease and two candidates were ineligible due to thyroid dysfunction. The enrolment protocol is shown in **Figure 1**.

### Baseline features of the participants

Baseline features of the participants were collected at diagnosis of T2DM prior to pharmacologic treatment. This study included 168 participants who were divided into two therapeutic cohorts according to their prescription of antihypertensive medications at study enrolment.

As shown in **Table 1**, baseline features including age (60.9 years vs. 62.4 years, P = 0.426), sex (49.4% female vs. 56.8% female, P = 0.339), serum HbA1c (8.54% vs. 8.17%, P = 0.183), body weight (69 kg vs. 72.9 kg, P = 0.11), systolic blood pressure (149 mm Hg vs. 147 mm Hg, P = 0.208), body mass index (BMI) (28.6 kg/m^2^ vs. 27.7 kg/m^2^, P = 0.265), and waist circumference (83 cm vs. 84 cm, P = 0.106) were similar between both cohorts. Moreover, laboratory tests demonstrated comparable kidney and liver function as well as plasma lipid fractions between these cohorts.

**Table 1:** Baseline features of the participants at disease diagnosis.

Parameters	Antihypertensive group (*n* = 87)	Control group (*n* = 81)	*P*value
Age (years)	60.9 ± 12.7	62.4 ± 11.3	0.426
Sex (Female)	43 (49.4%)	46 (56.8%)	0.339
Serum HbA_1c_ (%)	8.54 ± 1.86	8.17 ± 1.71	0.183
Body weight (kg)	69.0 ± 14.4	72.9 ± 16.4	0.11
Body mass index (kg/m^2^)	28.6 ± 5.5	27.7 ± 5.0	0.265
Waist circumference (cm)	83 ± 5.0	84 ± 6.0	0.106
Systolic blood pressure (mm Hg)	149 ± 8.6	147 ± 10.4	0.208
Diastolic blood pressure (mm Hg)	86.7 ± 8.7	88.0 ± 9.1	0.331
Serum creatinine (mg/dL)	0.87 ± 0.3	0.88 ± 0.29	0.983
Serum alanine transaminase (U/L)	37.4 ± 25.9	32.9 ± 21.2	0.213
Metformin dose (mg per day)	1425 ± 421	1426 ± 434	0.99
Fasting plasma glucose level (mg/dL)	198 ± 11.0	201 ± 12.2	0.177
Plasma triglycerides (mg/dL)	152 ± 71.8	157 ± 82.4	0.672
Plasma HDL-C (mg/dL)	44.4 ± 10.8	45.1 ± 12.2	0.685
Plasma LDL-C (mg/dL)	107 ± 32.1	111 ± 35.6	0.448
Urinary albumin-to-creatinine ratio (mg/g)	90.5 ± 247	74.0 ± 170	0.613

Data are expressed as means with standard deviation of the mean for continuous variables and number (%) for categorical variables. Variables are compared between groups using Student’s independent t-test for continuous variables and Pearson’s χ^2^-test for categorical variables. HbA_1c_, glycosylated hemoglobin A_1c_; mm Hg, millimeters of mercury; kg, kilograms; m, meters; mg, milligrams; HDL-C, high-density lipoprotein cholesterol; LDL-C, low-density lipoprotein cholesterol.

### Comparing the efficacy of therapeutic regimens on metabolic parameters after 24 weeks

After 24 weeks of therapy, participants receiving additional antihypertensive medications demonstrated lower mean serum HbA1c (6.62% vs. 7.01%, P = 0.01) and fasting plasma glucose (122 mg/dL vs. 129 mg/dL, P < 0.001) compared to recipients of standard diabetes treatment. Moreover, participants receiving additional antihypertensive prescription had significantly lower systolic blood pressure (132 mm Hg vs. 143 mm Hg, P < 0.001) and diastolic blood pressure (78.9 mm Hg vs. 86.0 mm Hg, P <0.001) compared to recipients of standard diabetes therapy. Otherwise, the two groups harbored similar levels of albuminuria (98.7 mg/g creatinine vs. 118 mg/g creatinine, P = 0.639), body weight (66.8 kg vs. 70.4 kg, P = 0.126), BMI (27.7 kg/m2 vs. 26.8 kg/m2, P = 0.227), and waist circumference (78.7 cm vs. 80 cm, P = 0.1), plasma triglycerides (128 mg/dL vs. 134 mg/dL, P = 0.674), plasma HDL-C (44.5 mg/dL vs. 44.8 mg/dL, P = 0.887), and plasma LDL-C (83.8 mg/dL vs. 82.3 mg/dL, P = 0.704). These findings are summarized in **Table 2**.

**Table 2:** Comparing the effect of pharmacologic therapy on metabolic parameters after 24 weeks.

Parameters	Antihypertensive group (*n* = 87)	Control group (*n* = 81)	*P*value
Serum HbA_1c_ (%)	6.62 ± 0.85	7.01 ± 1.1	0.01
Body weight (kg)	66.8 ± 13.8	70.4 ± 15.9	0.126
Body mass index (kg/m^2^)	27.7 ± 5.3	26.8 ± 4.8	0.227
Waist circumference (cm)	78.7 ± 4.8	80 ± 5.5	0.1
Fasting plasma glucose level (mg/dL)	122 ± 12.9	129 ± 8.64	<0.001
Plasma triglycerides (mg/dL)	128 ± 74.1	134 ± 83.2	0.674
Plasma HDL-C (mg/dL)	44.5 ± 11.1	44.8 ± 12.0	0.887
Plasma LDL-C (mg/dL)	83.8 ± 24.1	82.3 ± 26.7	0.704
Systolic blood pressure (mm Hg)	132 ± 7.8	143 ± 10.1	<0.001
Diastolic blood pressure (mm Hg)	78.9 ± 7.9	86.0 ± 9.1	<0.001
Urinary albumin-to-creatinine ratio (mg/g)	98.7 ± 214	118 ± 310	0.639

Data are expressed as means with standard deviation of the mean for continuous variables and number (%) for categorical variables. Variables are compared between groups using independent t-test for continuous variables and Pearson’s χ^2^-test for categorical variables. HbA_1c_, glycosylated hemoglobin A_1c_; mm Hg, millimeters of mercury; kg, kilograms; m, meters; mg, milligrams; HDL-C, high-density lipoprotein cholesterol; LDL-C, low-density lipoprotein cholesterol.

### Comparison of clinical parameters during pharmacologic intervention

As demonstrated in **Table 3**, both antidiabetic therapy and lifestyle changes significantly lowered serum HbA1c, plasma lipids, and blood pressure in both study cohorts after 24 weeks. Furthermore, participants demonstrated modestly reduced BMI and waist circumference at the follow-up clinic appointment presumably related to lifestyle changes.

**Table 3:** Comparison of clinical parameters during diabetes therapy.

Clinical parameters	Antihypertensive group (*n* = 87)	Control group (*n* = 81)
Serum HbA_1c_ (%)		
0 week	8.54 ± 1.86	8.17 ± 1.71
24 weeks	6.62 ± 0.85	7.01 ± 1.1
*P* value	<0.001	<0.001
Fasting plasma glucose level (mg/dL)		
0 week	198 ± 11.0	201 ± 12.2
24 weeks	122 ± 12.9	129 ± 8.64
*P* value	<0.001	<0.001
Plasma triglycerides (mg/dL)		
0 week	152 ± 71.8	157 ± 82.4
24 weeks	128 ± 74.1	134 ± 83.2
*P* value	<0.001	<0.001
Plasma LDL-C (mg/dL)		
0 week	107 ± 32.1	111 ± 35.6
24 weeks	83.8 ± 24.1	82.3 ± 26.7
*P* value	<0.001	<0.001
Plasma HDL-C (mg/dL)		
0 week	44.4 ± 10.8	45.1 ± 12.2
24 weeks	44.5 ± 11.1	44.8 ± 12.0
*P* value	0.918	0.75
Systolic blood pressure (mm Hg)		
0 week	149 ± 8.6	147 ± 10.4
24 weeks	132 ± 7.8	143 ± 10.1
*P* value	<0.001	<0.001
Diastolic blood pressure (mm Hg)		
0 week	86.7 ± 8.7	88.0 ± 9.1
24 weeks	78.9 ± 7.9	86.0 ± 9.1
*P* value	<0.001	<0.001
Urinary albumin-to-creatinine ratio (mg/g)		
0 week	90.5 ± 247	74.0 ± 170
24 weeks	98.7 ± 214	118 ± 310
*P* value	0.761	0.031
Body mass index (kg/m^2^)		
0 week	28.6 ± 5.5	27.7 ± 5.0
24 weeks	27.7 ± 5.3	26.8 ± 4.8
*P* value	<0.001	<0.001
Waist circumference (cm)		
0 week	83 ± 5.0	84 ± 6.0
24 weeks	78.7 ± 4.8	80 ± 5.5
*P* value	<0.001	<0.001

Data are expressed as means with standard deviation of the mean for continuous variables. Variables are compared to baseline levels using paired sample t-test. HbA_1c_, glycosylated hemoglobin A_1c_; mm Hg, millimeters of mercury; kg, kilograms; m, meters; mg, milligrams; HDL-C, high-density lipoprotein cholesterol; LDL-C, low-density lipoprotein cholesterol.

### Multiple regression analysis of clinical determinants of serum HbA1c levels

As shown in **Table 4**, multiple regression analysis reveals that both systolic blood pressure (regression coefficient: 0.0195, *P* = 0.0164) and diastolic blood pressure (regression coefficient: 0.021, *P* = 0.0252) are correlated with serum HbA_1c_ levels during diabetes treatment. In contrast, other clinical parameters had no significant influence on serum HbA_1c_ levels in this study.

**Table 4:** Multiple regression analysis of parameters that correlate with serum HbA1c levels.

Clinical parameters	Regression coefficient	*P*value
Plasma triglycerides (mg/dL)	0.001	0.364
Plasma HDL-C (mg/dL)	0.3	0.971
Systolic blood pressure (mm Hg)	0.0195	0.0164
Diastolic blood pressure (mm Hg)	0.021	0.0252
Body weight (kg)	0.01	0.06

HDL-C, high-density lipoprotein cholesterol; LDL-C, low-density lipoprotein cholesterol.

### Analysis of the association between antihypertensive therapy and serum glycosylated hemoglobin levels

**Figure 2:**
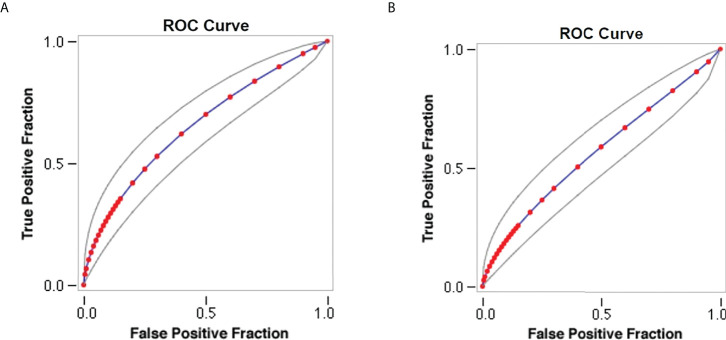
Sensitivity analysis of study parameters. **(A)** Model of antihypertensive medication and serum HbA1c levels. **(B)** Model of systolic blood pressure and serum HbA1c levels.

Sensitivity analysis demonstrates an association between antihypertensive medication and lower serum HbA1c levels with an area under the curve (AUC) of 0.7 as shown in **Figure 2A**. Moreover, sensitivity analysis observes a correlation between systolic blood pressure and serum HbA1c levels with an AUC of 0.64 as shown in **Figure 2B**.

## Discussion

In this study, stringent blood pressure management with the antihypertensive medication Amlodipine for 24 weeks significantly reduced mean serum HbA1C levels compared with standard diabetes therapy in patients with T2DM. Moreover, concomitant Amlodipine prescription lowered blood pressure with neutral effect on lipid profile and urinary albumin excretion. Sensitivity analysis demonstrates that antihypertensive prescription is associated with lower serum HbA1c levels and that systolic blood pressure correlates with serum HbA1c levels.

An antihypertensive drug may improve glycemic control by reducing superoxide dismutase activity, which is an established contributor to insulin resistance (16). Another study reported that two thirds of the patients attending hypertension clinics demonstrated insulin resistance (17), and antihypertensive medications can ameliorate oxidative stress in people with diabetes (18). *In vitro* evidence implies that Amlodipine improves insulin sensitivity by increasing the cross-sectional area of capillaries that supply glucose-metabolizing tissues (19). Furthermore, Amlodipine can attenuate the production of superoxide metabolites that impair glucose metabolism in a hyperglycemic milieu (20).

The finding that Amlodipine in addition to standard diabetes therapy improves glycemic control has therapeutic implications. Firstly, people with diabetes and inadequate glycemic control under metformin-based regimens may benefit from the addition of antihypertensive drug to help them attain euglycemia. Secondly, given the appreciable prevalence of concomitant hypertension in T2DM (21), medications that lower blood pressure not only reduce vascular complications but also improve glycemic control in at-risk patients. Moreover, since Amlodipine is cost-effective with an excellent safety profile (22), clinicians may consider its prescription in people with diabetes and concomitant hypertension before embarking on an extensive adjustment of antidiabetic regimens. Finally, since metformin is a first-line antidiabetic medication endorsed by endocrine societies (23), the finding that Amlodipine amplifies its glucose-lowering effect may be applicable to other metformin-based regimens.

Moreover, other antihypertensive drugs including renin-angiotensin-aldosterone system (RAAS) inhibitors exert favorable effects on glucose metabolism (24). Clinical studies have shown that RAAS inhibition attenuates the development of diabetes in people with hypertension (25, 26). Positive effects of RAAS inhibitors on glucose metabolism may involve systemic vasodilation, which increases the perfusion of glucose-metabolizing tissues such as the skeletal muscle (27), as well as reduction of reactive oxygen species that interfere with glucose oxidation (28). In contrast, diuretics or beta-adrenoceptor blockers exhibit negative effects on the metabolic profile (29). Therefore, glucose metabolism may become an issue when prescribing antihypertensive medications for people with T2DM.

This study has several advantages. Firstly, participants in the study received solely the antidiabetic drug metformin, which avoids the confounding effect of second-line antidiabetic drugs on glycemic control. Secondly, participants in both therapeutic groups were stringently selected with comparable baseline features at study enrolment. Thirdly, patients received comprehensive nutrition consultation by certified diabetes educators to ensure compliance with prescriptions and lifestyle modification.

Nonetheless, several limitations arise from the study protocol. Firstly, a protocol of shared decision making was applied in terms of antihypertensive drug prescription, and this non-randomized design can lead to potential selection bias (30). This investigation was intended as a pilot study and further randomized studies are required to confirm the relevant findings. In addition, this study has a relative shorter follow up time of 24 weeks, which may not reflect long term changes in clinical parameters. Thirdly, improvements in glycemic control are influenced not only by pharmacologic intervention but also by other lifestyle changes such as exercise, which investigators had limited oversight in this study.

## Conclusions

Antihypertensive treatment with Amlodipine in addition to standard diabetes therapy improves glycemic control in patients with T2DM. Therefore, antihypertensive therapy may be an appropriate option in people with diabetes and concomitant hypertension to help maintain euglycemia. Further studies are indicated to determine whether other classes of antihypertensive drugs also enhance glycemic control in T2DM.

## Data availability statement

The original contributions presented in the study are included in the article/supplementary material. Further inquiries can be directed to the corresponding author.

## Ethics statement

The studies involving human participants were reviewed and approved by Institutional Review Board of Changhua Christian Hospital (Ethics committee identifier: P201411-15). The patients/participants provided their written informed consent to participate in this study.

## Author contributions

J-CL: drafting the manuscript, revising the manuscript. P-CC: conceptualization, data analysis, revising the manuscript. C-NH: conceptualization, revising the manuscript. L-FJ: revising the manuscript. Y-SW (equal contribution as first author): drafting the manuscript, revising the manuscript. C-LL: conceptualization, project design, revising the manuscript. All authors contributed to the article and approved the submitted version.

## Conflict of interest

The authors declare that the research was conducted in the absence of any commercial or financial relationships that could be construed as a potential conflict of interest.

## Publisher’s note

All claims expressed in this article are solely those of the authors and do not necessarily represent those of their affiliated organizations, or those of the publisher, the editors and the reviewers. Any product that may be evaluated in this article, or claim that may be made by its manufacturer, is not guaranteed or endorsed by the publisher.
